# Upregulate KIF4A Enhances Proliferation, Invasion of Hepatocellular Carcinoma and Indicates poor prognosis Across Human Cancer Types

**DOI:** 10.1038/s41598-017-04176-9

**Published:** 2017-06-23

**Authors:** Guojun Hou, Chuanpeng Dong, Zihui Dong, Gang Liu, Huilin Xu, Lei Chen, Lei Liu, Hongyang Wang, Weiping Zhou

**Affiliations:** 1 The Third Department of Hepatic Surgery, Third Affiliated Hospital of Second Military Medical University, Shanghai, 200438 China; 2 National Center for Liver Cancer, Shanghai, 201805 China; 3 Institutes of Biomedical Sciences, Fudan University, 200032 Shanghai Shi, China

## Abstract

Hepatocellular carcinoma (HCC) is one of the most aggressive and heterogeneous cancers worldwide. Herein, we demonstrate KIF4A (Chromosome-associated kinesin KIF4A) as a potential biomarker, is up-regulated in most samples of HCC. The expression level of KIF4A in tumor tissue is significantly associated with the survival time, and a significant correlation between KIF4A expression and clinical information stage, metastasis and tumor dimension was observed. We further measured the proliferation and migration ability of two HCC cell lines, HCC-LM3 and PLC/PRF/5, following KIF4A-siRNA transfection. Knocking down of KIF4A significantly reduced migration and proliferation ability. Moreover, we also measured the proliferation and migration ability of two HCC cell lines through KIF4A overexpression, and found that KIF4A overexpression could enhance migration and proliferation ability, indicating that KIF4A exhibits oncogenic effects. Besides, study based on TCGA cohorts also reveals high KIF4A mRNA expression are significantly associated with shorter overall survival in multiple cancer types. Gene sets enrichment analysis exhibited that cell cycle related pathways and p53 signaling pathways to be top altered pathways of in KIF4A-high expression group in HCC, suggesting the potential role of KIF4A in mediating tumor initiation and progression. In summary, our work identified KIF4A as a potential predictive and prognostic marker for hepatocellular carcinoma.

## Introduction

Hepatocellular carcinoma (HCC) is one of the most common cancers and is the leading causes of cancer-related death worldwide^1^. The rapid recurrence of 70% to 100% patient after resective surgery contributes to the majority of short survival of HCC^2^. The heterogeneity, aggressive nature and the lack of general consensus treatment render it difficult to develop effective therapies for HCC^3^. Thus it raises the need to clarify the molecular mechanism of HCC, which may hold the promise of more biomarker for HCC diagnosis, prognosis and target therapy.

Kinesins are molecular motors proteins that move along microtubule tracks to support their multiple functions in intracellular transport or cell division^4^. Previous studies also have indicated kinesins play critical roles in several malignancies, including tumoral development and progression^5, 6^. Among them, KIF4A (Chromosome-associated kinesin KIF4A) was identified as an oncogene and a contributor to malignant progression in lung cancer^7^, oral cancer^8^ and breast cancer^9^. Besides, KIF4A is reported amplified and elevated in cervical cancer in a microarray study^10^. Gao *et al*. have reported that up-regulation of KIF4A inhibits stomach cancer cell growth^11^. However, the potential molecular evidence and mechanisms of KIF4A in HCC remain to be explored.

In this study, we demonstrated KIF4A may be a potential diagnostic and prognostic marker in HCC. Comparative analysis revealed that KIF4A correlated with important clinicopathological features of HCC. Functional studies of KIF4A demonstrated that knockdown/overexpression KIF4A could inhibit/promote proliferation and migration of HCC cell. We further evaluated the prognostic significance of KIF4A expression across human cancer types by utilizing The Cancer Genome Atlas (TCGA) database. Higher KIF4A mRNA expression is significantly associated with poor outcome in multiple cancer types. Gene sets enrichment analysis revealed that cell cycle related pathways were altered mostly under the changes of KIF4A expression.

## Results

### KIF4A is up-regulated in HCC

**Figure 1:**
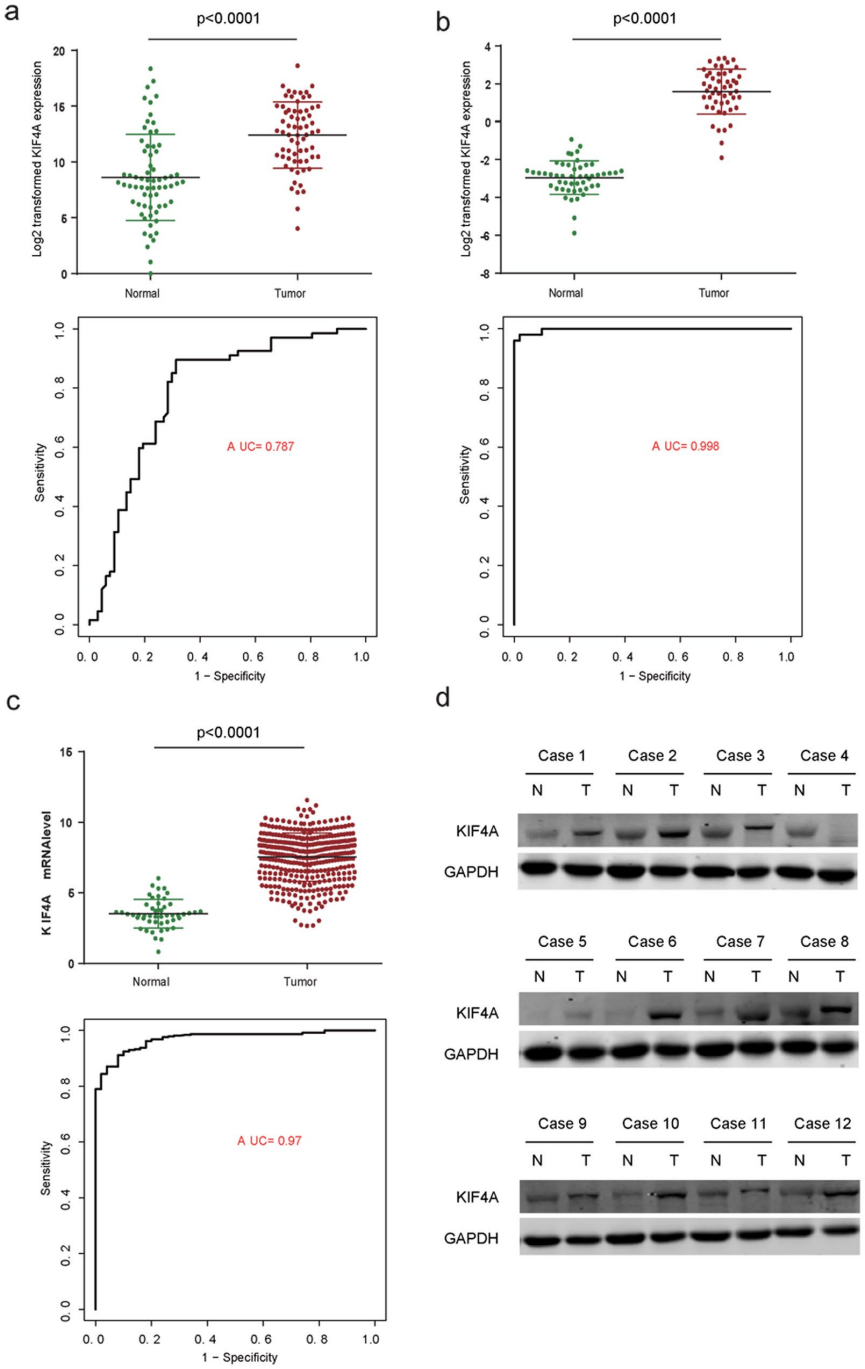
The diagnostic effect of KIF4A in HCC. The mRNA expression level of KIF4A is up-regulated in tumor tissues compared to the normal in (**a**, top panel) Real time PCR in 67 pair tumor vs normal tissues (**b**, top panel). RNA sequence of 50 pair tumor vs normal tissues and (**c**, top panel) RNA-seq of unpaired tumor vs normal tissues in TCGA liver cancer; respectively, the diagnostic effect of KIF4A were evaluated by ROC curve in three datasets (**a**–**c**, bottom panel). The protein expression level of KIF4A is up-regulated in tumor tissues compared to the normal in (**d**).

RT-PCR was used to evaluate mRNA expression levels of KIF4A upon 67 solid tumor tissues and corresponding normal liver tissues (clinical information of patients is available in Supplementary Table S1), the melting curve was shown in Supplementary Figure 1. We found that KIF4A is significantly elevated in HCC tumors compared to their corresponding normal tissues (Fig. 1a, top panel). These findings were validated in a validation cohort of 50 pairs of normal-tumor samples (Fig. 1b, top panel) according to RNA-seq analysis (GSE77314). KIF4A expression level also retracted from TCGA liver cancer cohorts for external validation, and KIF4A expression was shown significantly increased in hepatocellular carcinoma tissue compared with normal liver tissue (Fig. 1c, p < 0.001, top panel). Western blot assays showed that the protein level of KIF4A was also higher in HCC tumor tissues than in their matched counterparts (Fig. 1d). These results demonstrated that KIF4A was overexpressed in HCC.

Receiver operating characteristic (ROC) analysis was performed to assess the sensitivity and specificity of the diagnostic utility of KIF4A in these three datasets. Then area under the curve (AUC) values were measured based on ROC curves. The AUC reached 0.787, 0.998 and 0.97 in the RT-PCR cohort, RNA-seq cohort and TCGA cohort, respectively (Fig. 1a–c, bottom panel), indicating that it is a potential valuable biomarker for the diagnosis of HCC.

### KIF4A is a prognostic marker in HCC

**Figure 2:**
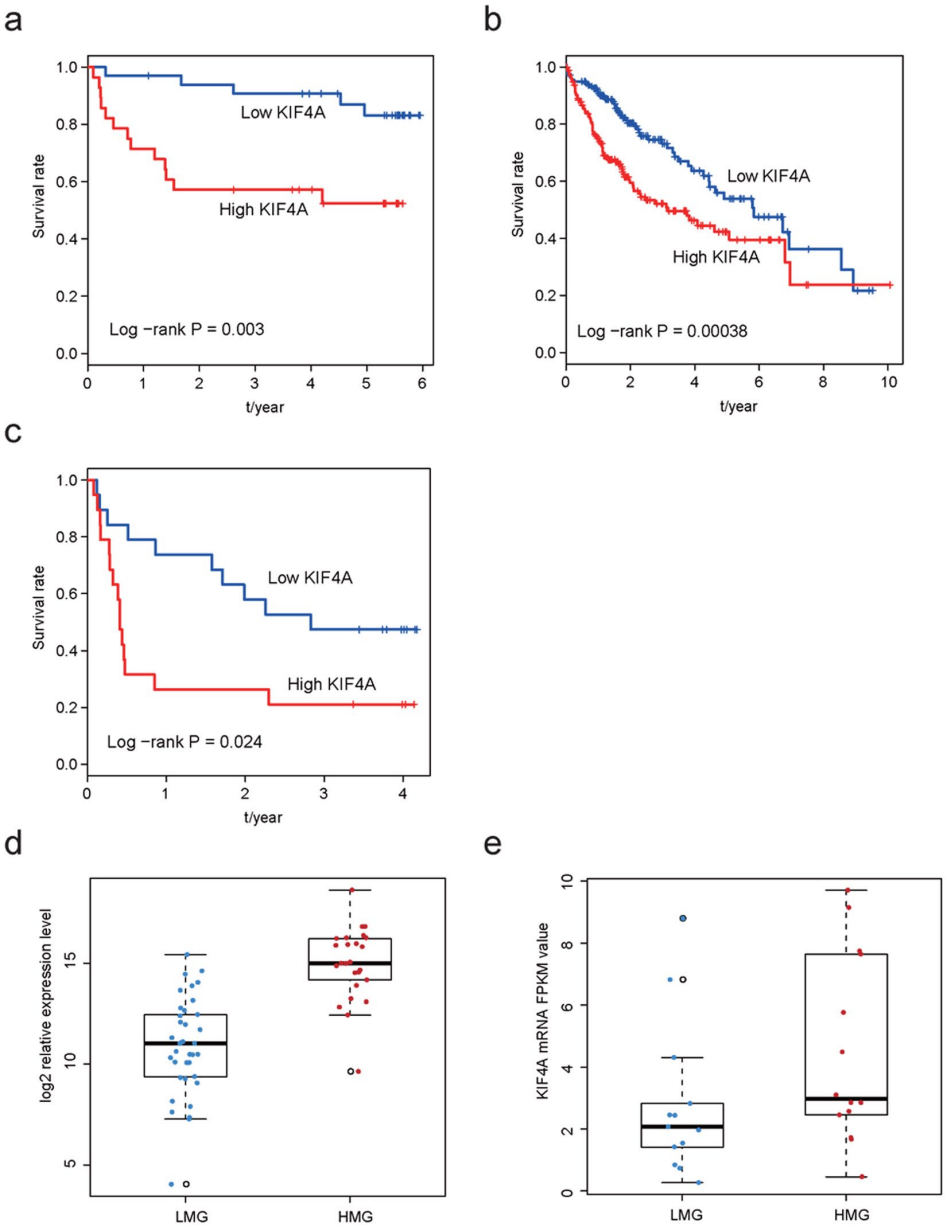
The prognostic effect of KIF4A in HCC. Patients with high KIF4A expression has a relatively poor overall survival rate according result from RT-PCR (**a**), TCGA (**b**) and GSE77314 (**c**); the mRNA expression level of high metastasis patients is significantly higher compared with low metastasis patients in our RT-PCR cohort (**d**) and GSE77314 cohort (**e**). FPKM: Fragments Per Kilobase of transcript per Million mapped reads.

The association between KIF4A expression and HCC patients’ prognosis were further evaluated by Kaplan-Meier survival analysis and log-rank test. We analyzed the survival status of patients with high and low expression of KIF4A based on 67 RT-qPCR results (by using the median KIF4A expression level as the cutoff point, and the same method was performed on GSE77314 and TCGA datasets). Patients with high expression of KIF4A had significantly shorter overall survival time than patients with low expression (log-rank P value = 0.003) (Fig. 2a). Similar to the findings from the RT-PCR cohort, patients with the high expression KIF4A in TCGA (N = 371) and GSE77314 (N = 50) had shorter survival time than patients with low expression KIF4A, and log-rank P to be 0.00038 and 0.024 (Fig. 2b,c), respectively, confirming the prognostic utility of KIF4A in HCC. We then evaluated the expression of KIF4A in 37 low metastasis patients and 25 high metastasis patients from RT-PCR datasets, and the highest expression of KIF4A was observed in high metastasis patients group (Fig. 2d). Result of GSE77314 datasets exhibited similar association between high KIF4A expression and high metastasis (Fig. 2e), indicating the possible pro-metastatic role of KIF4A.

### The correlations between KIF4A expression and clinical information

To characterize the role of KIF4A in HCC, we analyzed the correlation between clinical characteristics and the KIF4A gene expression in two independent datasets (QPCR and GSE77314). As shown in Table 1, intrahepatic metastasis was detected in the KIF4A-low group with 4.4% (1/23), while 35% (8/23) in KIF4A-high group (p = 0.022). It is also the case in the validation dataset (14% vs 52%, p = 0.0097). The proportion of relative larger tumor (dimension of primary tumor >5 cm) reached 62% (21/34) in KIF4A-high group while only 30% in KIF4A-low group. The validation dataset also confirmed this result. In addition, we found that recurrence rate, primary tumor stage, and daughter nodules were also significantly different in the discover dataset, while the validate dataset showed no significant difference. This may probably due to the limited sample size of validate dataset. These results indicate that KIF4A was associated with clinicpathological information.

**Table 1:** The association between KIF4A expression and clinical information.

Variable	Real time PCR cohort			GSE77314 cohort		
	low KIF4A	high KIF4A	p Value	low KIF4A	high KIF4A	p Value
Recurrence			**0**.**0123**			0.5231
No	21	10		10	7	
Yes	4	12		10	12	
Metastasis			**0**.**022**			**0**.**0097**
No	22	15		19	10	
Yes	1	8		3	11	
Age			0.5896			1
<60	23	26		19	20	
≥60	10	8		6	5	
Gender			0.6135			1
Male	31	33		4	3	
Female	2	1		21	22	
Cirhosis			0.3873			1
No	6	10		17	18	
Yes	23	21		7	6	
T Stage			**<0**.**0001**			0.1284
1-2	31	16		20	14	
3-4	1	18		5	11	
Diameter			**0**.**0143**			**0**.**0421**
≤5	23	13		14	6	
>5	10	21		11	19	
Daughter nodule		**0**.**0029**			0.3772	
No	31	23		18	14	
Yes	1	11		7	11	
BCLC			0.0537			0.1963
A-B	32	29		21	16	
C-D	0	5		4	9	

The KIF4A-low and KIF4A-high group is classified according to the median expression level of KIF4A. *Some clinical information is missing.

### KIF4A effected the proliferation and migration of HCC cell lines

**Figure 3:**
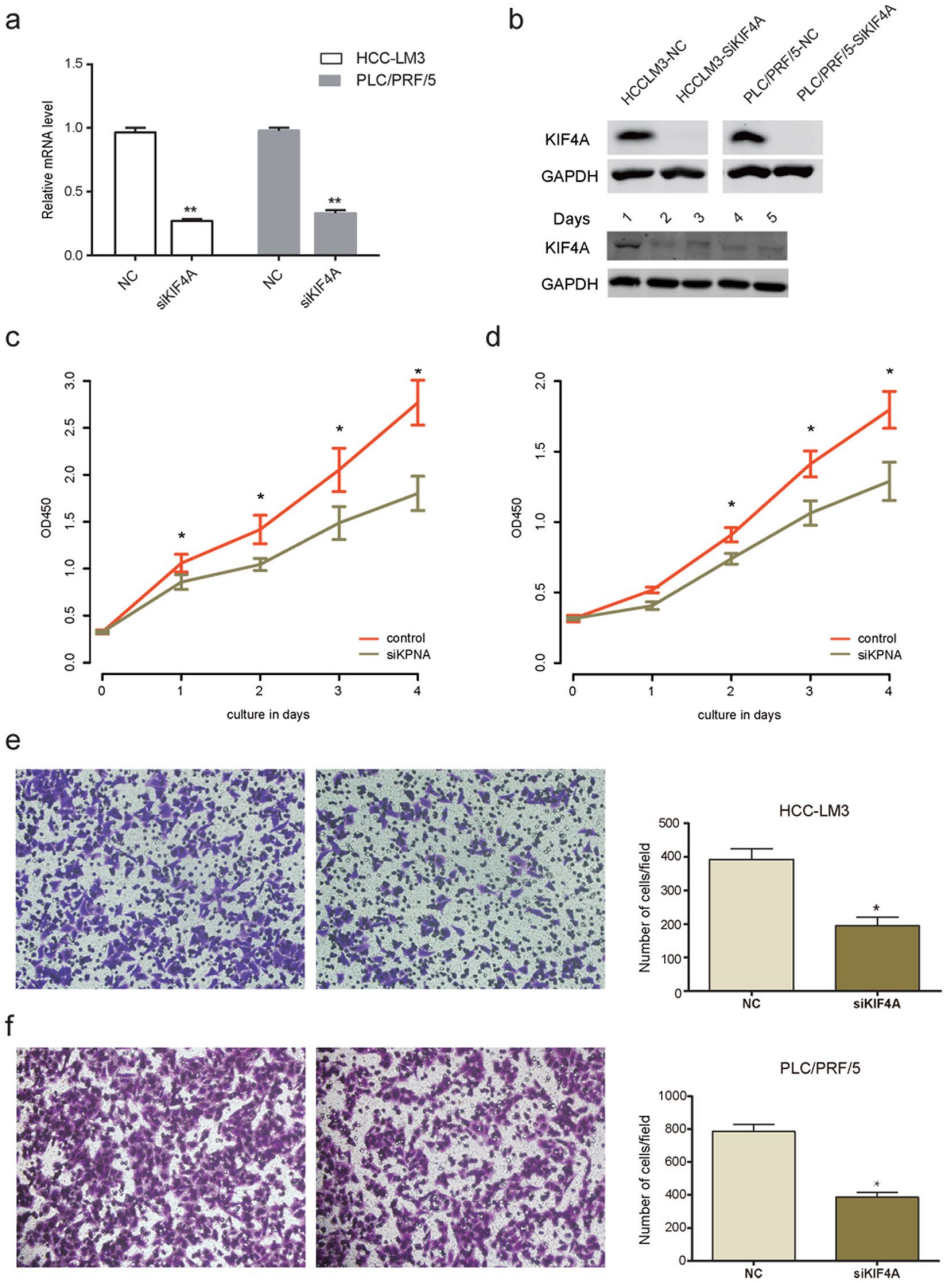
The proliferation and migration promoting effect of KIF4A in HCC cell lines. KIF4A expression was suppressed by RNAi in mRNA (**a**) and protein (**b**) level (upper panel, KIF4A protein expression in HCC-LM3 and PLC/PRF/5 cell lines; lower panel, each time point protein expression of KIF4A in HCC-LM3 cell line); KIF4A knockdown decreased cell proliferation rate of HCC-LM3 (**c**) and PLC/PRF/5 (**d**); KIF4A knockdown impaired migration rate in HCC-LM3 (**e**) and PLC/PRF/5 (**f**).

To evaluate the function of KIF4A *in vitro*, we firstly silenced KIF4A using siRNAs upon two different HCC cell lines, HCC-LM3 and PLC/PRF/5. The efficiency of the knockdown of KIF4A expression was examined by real-time PCR and western blotting (Fig. 3a,b). Knockdown of KIF4A significantly reduced OD450 value compared to their corresponding control cell lines, both in HCC-LM3 and PLC/PRF/5 (Fig. 3c,d). Moreover, to further explore the relationship between KIF4A expression and the proliferation capacity of HCC, we constructed KIF4A overexpression plasmids (Supplementary Figure 2, top panel). And the overexpression of KIF4A resulted in increased proliferation rate (Supplementary Figure 2, bottom panel), which demonstrated that KIF4A could affect proliferation rate of HCC cells.

Our previous results demonstrated that the elevated KIF4A was associated with metastasis in HCC. Therefore, we evaluated the impact of KIF4A expression on cell migration ability. Following siRNA-mediated suppression of KIF4A for 48 hours, the number of migrated cells of control group was 2-folds higher than that observed in the KIF4A knock down group in both HCC-LM3 and PLC/PRF/5 (Fig. 3e,f). In addition, the overexpression of KIF4A resulted in enhanced migration ability (Supplementary Figure 3), indicating that KIF4A could affect metastasis of HCC cells too.

### KIF4A has prognosis effect in multiple human cancer types

**Figure 4:**
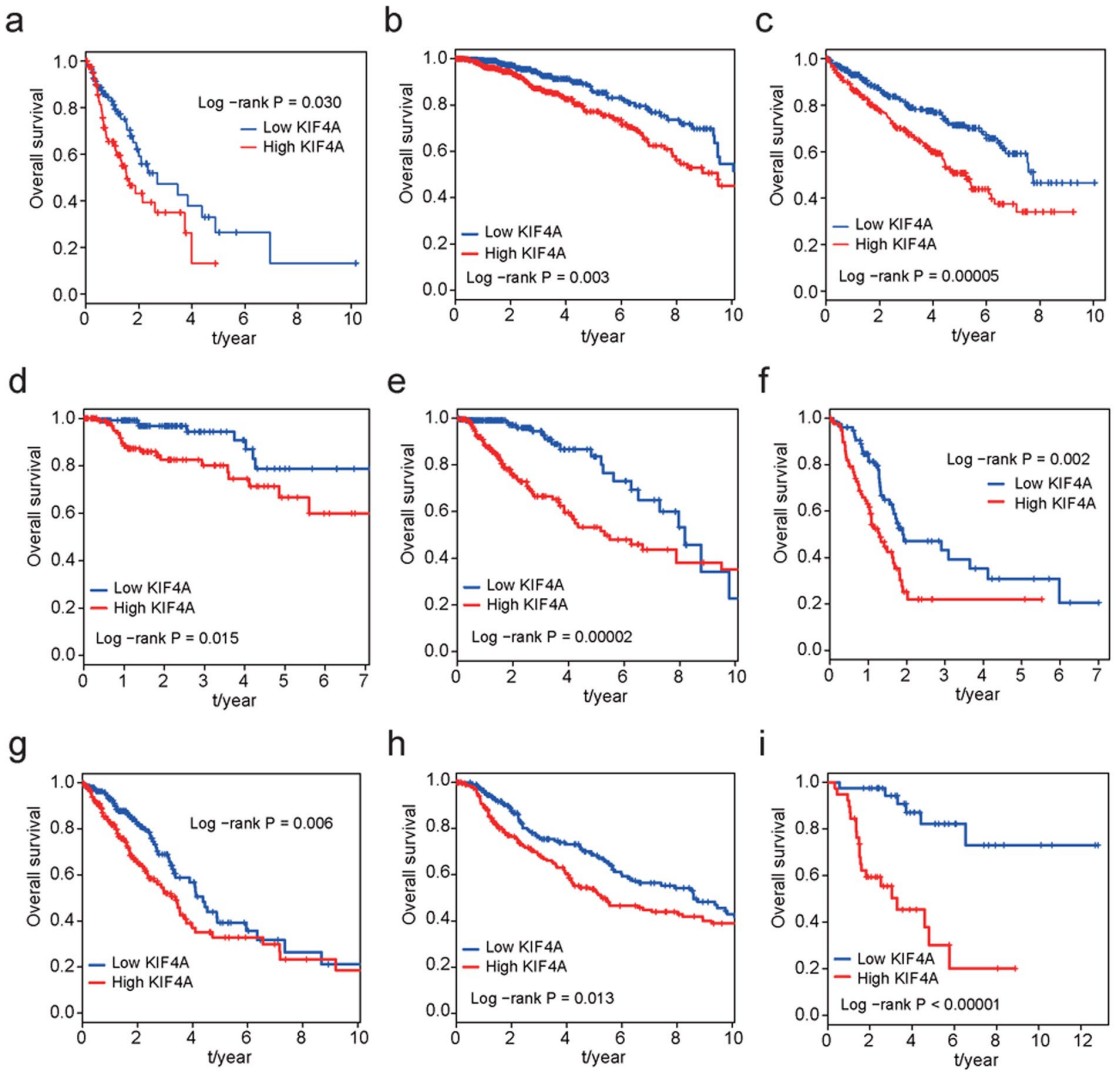
Prognosis effect of KIF4A expression level on overall survival in other human cancer types. (**a**) Esophageal carcinoma, (**b**) Breast cancer, (**c**) Kidney renal clear cell carcinoma, (**d**) Kidney renal papillary cell carcinoma, (**e**) Brain lower grade glioma, (**f**) Pancreatic adenocarcinoma, (**g**) Lung adenocarcinoma, (**h**) Skin cutaneous melanoma, and (**i**) Adrenocortical carcinoma.

Kaplan-Meier analyses were used on the TCGA cohorts to assess the prognostic value of KIF4A expression among different human cancer types (Supplementary Tables S1 and S2). We found that higher levels of KIF4A mRNA were correlated with shorter overall survival in other 9 human cancer types (Fig. 4a–i), which is consistent with studies performed by other researchers. Particularly, esophageal carcinoma (Fig. 4a), breast invasive carcinoma (Fig. 4b), kidney renal clear cell carcinoma (Fig. 4c), low grade gliomas (Fig. 4e) showed larger difference on overall survival between high and low levels of KIF4A expression. Along with our results on HCC, these findings strengthen the proposal that KIF4A may function as a general oncogene in multiple human cancer types.

### Potential role of KIF4A in malignant carcinogenesis and metastasis

**Figure 5:**
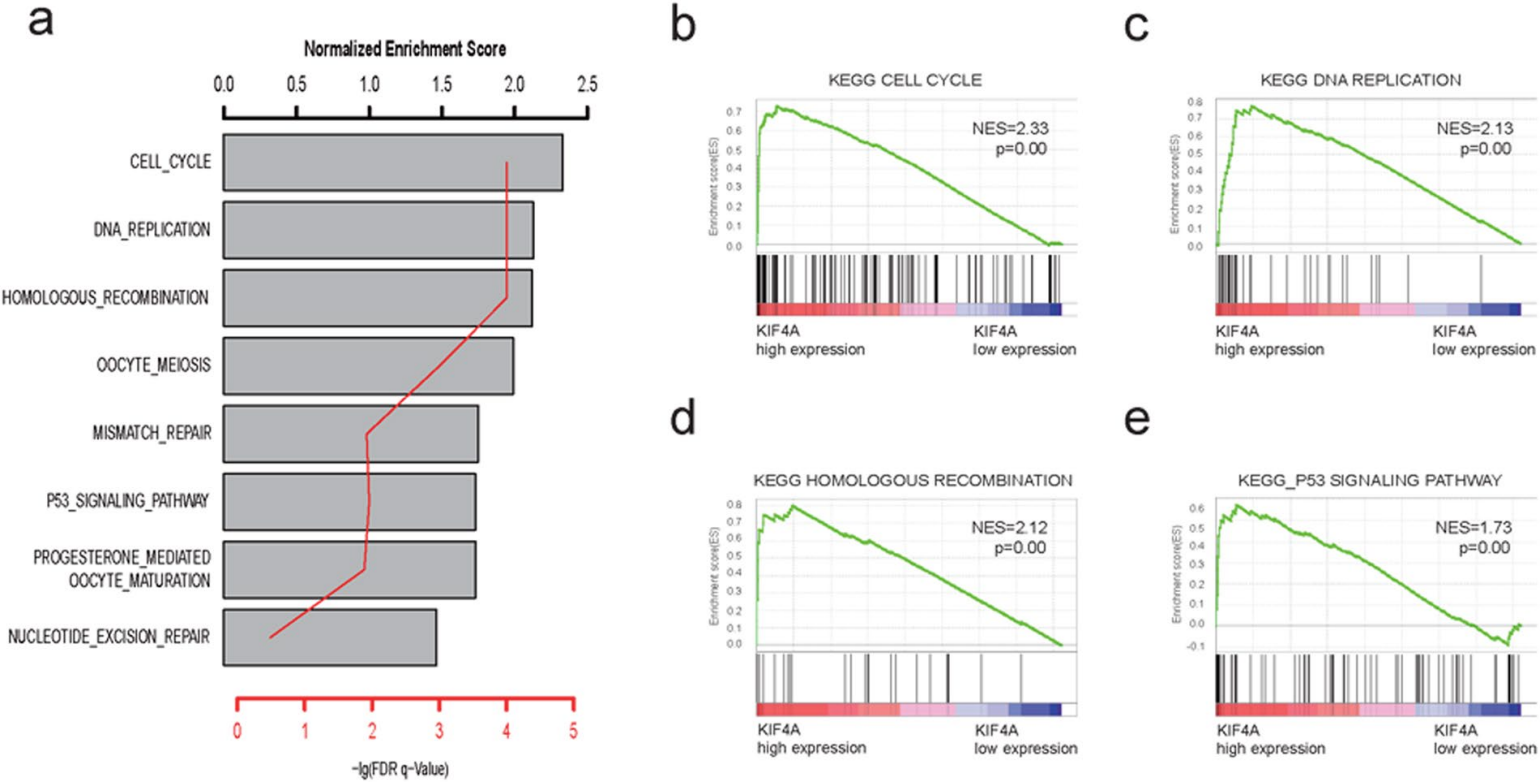
Gene sets enrichment analysis delineates associated with KIF4A expression level in the TCGA liver cancer cohort. Several cancer related signaling pathways were significantly enriched in the high-KIF4A group (**a**), such as cell cycle (**b**), DNA replication (**c**), homologous recombination (**d**) and p53 signaling (**e**) pathway. Normalized enrichment score (NES) is used to evaluate enrichment result. The –log p indicates the -log 10 transformed FDR p value of the corresponding pathway.

To further investigate the potential signaling pathway which contributes to KIF4A-mediated proliferation and migration of HCC cells, gene set enrichment analysis(GSEA) was used to find out associated signaling pathways. We compared the gene expression profile of patients with high KIF4A expression and those with low KIF4A expressing in TCGA liver cancer cohort (total 371 patients, divided by the median KIF4A mRNA expression value). Among the 186 curated KEGG gene sets, we found several cancers related pathways were significantly enriched in the high-KIF4A group (Fig. 5a). Of these pathways, we noticed that cell cycle (Fig. 5b), DNA replication (Fig. 5c), and homologous recombination (Fig. 5d) was significantly enriched and most genes enriched in these pathway was up-regulated in KIF4A highly expressed group. Taking together with significantly enrichment of p53 signaling pathway (Fig. 5e), our result suggested that KIF4A may promote HCC cell growth and metastasis by mediating cell cycle related and p53 signaling pathways.

## Discussion

Kinesins are a superfamily of motor proteins that participate in mitosis, intracellular transportation and cytoskeletal reorganization^12^. Alterations of kinesins play essential roles in cancer cell proliferation, invasion and metastasis^13, 14^. In present study, we evaluated the expression level of KIF4A in RT-PCR, GSE77314 and TCGA HCC cohorts. We found that KIF4A might be a potential diagnostic marker for distinguishing normal and tumor tissues, and KIF4A mRNA expression in tumor tissues were elevated in all cohorts, with AUC reaching 0.787, 0.998 and 0.97 respectively. The expression level of KIF4A was demonstrated as a HCC prognostic marker, and presented association with specific clinicopathological features of HCC. Further, we functionally validated the effect of KIF4A expression, demonstrating that knockdown of KIF4A leads to reduced proliferation and migration. In addition, the prognostic significance of KIF4A expression in other cancer types was validated in TCGA cohorts.

Earlier reports of the potential prognosis effect of KIF4A in breast cancer and lung cancer were confirmed in present study using TCGA cancer cohorts. Our result also indicates increased KIF4A mRNA expression significantly associated with short survival in other nine cancer type, which suggested KIF4A may play essential oncogenic role of in various malignant cancer progression. To further investigate the potential mechanism underlying KIF4A-mediated migration and proliferation in HCC, KEGG pathway enrichment analysis was carried out to evaluate gene expression difference between KIF4A-high and KIF4A -low groups. Our results indicated that the top significantly affected pathways in HCC were the cell cycle, mitosis related pathways and p53 signaling pathway, thus supporting our *in vitro* findings. Previous study demonstrated KIF4A plays an essential role in midzone formation and cytokinesis during the metaphase-anaphase transition^15^. By modulating BRCA2/Rad51 pathways, KIF4A function as an early response molecule involved in DNA damage response and repair response system^16^. According to a genome-wide microarray study, AURKB and KIF4A were identified as differently expressed genes in cases of aggressive recurrence of HCC^17^. KIF4A activity in anaphase spindle function could be controlled by Aurora B, which inhibits microtubule dynamics and restricts central spindle size by activating KIF4A locally^18^. This should be a potential reasonable mechanism of KIF4A in HCC and is consistent with our findings in KEGG pathways enrichment analysis. In addition, Zhu et l reported that hepatitis B virus can upregulate the expression of KIF4A in liver tissue^19^. This suggested KIF4A may mediate carcinogenic mechanisms of HBV in HCC.

In conclusion, our study demonstrated KIF4A as a novel diagnostic and prognostic marker. It promotes HCC cell proliferation and migration through cell cycle related pathways and thus may serve as a potential target for the HCC treatment in future.

## Materials and Methods

### Ethics statement

All the clinical specimens were obtained with informed consent and approved by the the

Clinical Research Ethics Committee of Eastern Hepatobiliary Surgery Hospital, Informed consent was obtained from all patients involved in this study. All the experiments were performed in accordance with the approved guidelines of the Institutional Research Ethics Committee of the Second Military Medical University University.

### Patients and Samples

In our study, 67 patients who underwent curative hepatectomy at the Eastern Hepatobiliary Surgery Hospital (Shanghai, China) from January 2009 to September 2012 were randomly selected for qRT-PCR analysis, and these tissues were aimed to investigate the mRNA level of KIF4A and the clinicopathologic characteristic was shown in Table 1.

### TCGA data processing

KIF4A mRNA expression levels and clinical information of TCGA study samples were obtained from Cancer Genomics Browser of University of California Santa Cruz (https://genome-cancer.ucsc.edu)^20^. TCGA patient and tissue sample characteristics are described in Supplementary Tables S1 and S2. Gene expression and clinical data of patients were integrated according to their barcode ID. Survival groups were classified according to the median expression level of each gene, and survival differences were evaluated using the R package “survival”.

### RNA extraction and Quantitative real-time PCR (RT-QPCR)

Informed consent was got from all patients whose tissue was used in this study. RNA was extracted with Trizol kit (Invitrogen, CA) according to standard manufacture provided protocol. The quality and quantity of isolated RNA was measured by Nanodrop 2000 (Thermo Scientific, USA), and cDNA was synthesized with 2 μg total extracted RNA by using random primers along with M-MLV Reverse Transcriptase (Invitrogen, CA). Expression level of KIF4A was quantified using real-time polymerase chain reaction (RT-PCR) using the primers, forward, 5′- TGAACTCCCAGTCGTCC-3′ and reverse, 5′-GCACTGATTACATTTCCC-3′, performed using SYBR Green PCR kit (Applied Roche, Switzeland) and ABI PRISM 7900 sequence detector (Applied Biosystems, Carlsbad, CA), with 18s as the endogenous control with the primers, forward, 5′- CGGCTACCACATCCAAGGAA-3′ and reverse, 5′-GCTGGAATTACCGCGGCT-3′. Each sample was tested in duplicate, and the relative mRNA expressions were determined based on the CT values and were normalized via the same endogenous reference 18s expression level.

### Cell culture and transfection

Liver cancer cell lines involved in this study including PLC/PRF/5 and HCC-LM3 were purchased from Cell Bank of Type Culture Collection of Shanghai Institute of Cell Biology, Chinese Academy of Sciences. All of the cell lines were used at previous passages^21–24^ and no morphological change or altered growth rate was observed during maintenance of the cultures. Cell lines were routinely cultured at 37 °C containing 5% CO2 in Dulbecco’s modified Eagle’s medium adding 10% fetal bovine serum. Cells were passed every 1–2d to maintain logarithmic growth.

For *in vitro* study, PLC/PRF/5, HCC-LM3 were transfected with siRNA (Biotend, Shanghai, People’s Republic of China) against KIF4A to knock down KIF4A, or plamid to increase the expression of KIF4A, siRNA-control and PcDNA3.1 were used as a negative control. The sense sequence is: UUAGAUGAUUAAGUUCAGC dTdT. The siRNA transfection was performed with INTERFERin reagents (Polyplus, France) according to the manufacturers’ instructions. Briefly, for each well (6 well, for example), 10 μl (20 μM storage concentration) siRNA duplexes were diluted into 200 μl of medium without serum and mixed by pipetting up and down. Then, 8 μl of INTERFERin was added into the 200 μl of siRNA duplexes, homogenized by vortex immediately for 10 seconds, incubated for 20 minutes at room temperature to allow transfection complexes to form between siRNA duplexes and INTERFERin. Then, 200 μl of transfection mix was added into the 1.8ml cell culture medium to complete a final concentration of 100 nM siRNA. Finally, cell culture medium was homogenized by gently swirling the plate. For plasmid transfection experiments, cells were transiently infected using PEI (Polyplus, France) as described previously^25^.

### Western blot

Total protein was extracted using RIPA Lysis Buffer and PMSF (Thermo Scientific, USA) according to the manufacturer provided protocols, and centrifuged at 12,000 rpm for 15 minutes. Total concentrations proteins were measured with standard bicinchoninic acid assay. Antibody dilutions were 1:500 for the KIF4A antibody (Abcam, Cambridge, MA) and 1:10000 for GAPDH (Santa Cruz Biotechnology) and immunocomplexes were incubated with thefluorescein-conjugated secondary antibody. Antibody binding was measured with Odyssey infrared scanner (Li-CorBiosciences, Inc.)

### Cell migration and proliferation analysis

Migration assays were performed using thetranswell filter chambers (Costar, Corning, NY) according to the manufacturer provided protocol. Totally, levitate 1 × 105 cells in serum-free medium, and added the medium onto the top of chamber. Ten percent FBS containing medium was added to the lower chamber. Following 12 hours of incubation, experimental cells on the lower surface of the chamber were photographed and counted using a microscope in six random fields per field for each group. These experiments were performed in triplicate. For proliferation assay, cells were planted into 96-well plates (3000 cells/well) and cell proliferation rate was measured by Cell Counting Kit-8 (Dojindo Laboratories, JA) every 24 hours.

### Data analysis

Survival differences among groups were estimated using the R package “survival”. Log-rank test was used to determine differences among survival groups according to KIF4A mRNA levels in HCC and other cancer types and visualized with Kaplan-Meier curve plot. The correlation relationship between clinical information and expression was calculated using fisher’s exact test. The receiving operating character (ROC) curve were calculated and drawn with R package “pROC”. Gene sets enrichment analysis was performed with GSEA software (http://www.broadinstitute.org/gsea) upon KEGG gene sets collection (c2.cp.kegg.v5.2, 186 gene sets)^26^. To enriched gene sets, a false discovery rate (FDR) value < 0.01 after performing 1,000 permutations were considered to be significantly.

## Electronic supplementary material

supplementary information
