# Supplementary material for: Upregulate KIF4A Enhances Proliferation, Invasion of Hepatocellular Carcinoma and Indicates poor prognosis Across Human Cancer Types
Source: Sci Rep. 2017 Jun 23;7:4148. doi: 10.1038/s41598-017-04176-9 (PMC5482880; doi:10.1038/s41598-017-04176-9)
Supplement: Supplementary file 1 — supplementary information [file 41598_2017_4176_MOESM1_ESM.pdf]

Upregulate KIF4A Enhances Proliferation, Invasion of Hepatocellular Carcinoma and Indicates poor prognosis Across Human Cancer Types

Guojun Hou[1,*], Chuanpeng Dong [3,*], Zihui Dong[2,*], Gang Liu[3,*], Huilin Xu[3], Lei Chen[2], Lei Liu[3, $], Hongyang Wang[2, $], Weiping Zhou[1, $]

[1]The Third Department of Hepatic Surgery, Third Affiliated Hospital of Second Military Medical University, Shanghai, 200438, China;

[2] National Center for Liver Cancer, Shanghai, 201805, China;

[3] Institutes of Biomedical Sciences, Fudan University, 200032;

*These authors have contributed equally to this work

$Corresponding authors:

Weiping Zhou: ehphwp3@126.com

Hongyang Wang:hywangk@vip.sina.com

Lei Liu: liulei_sibs@163.com

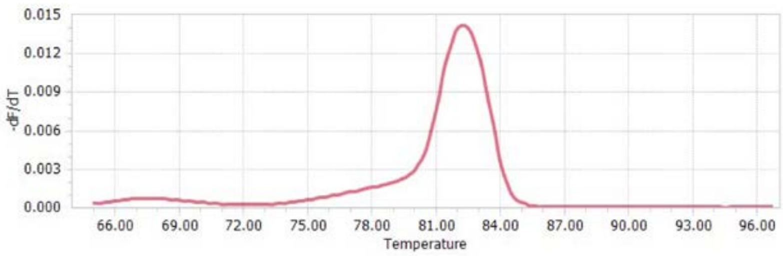

Supplementary Figure 1. The melting curve.

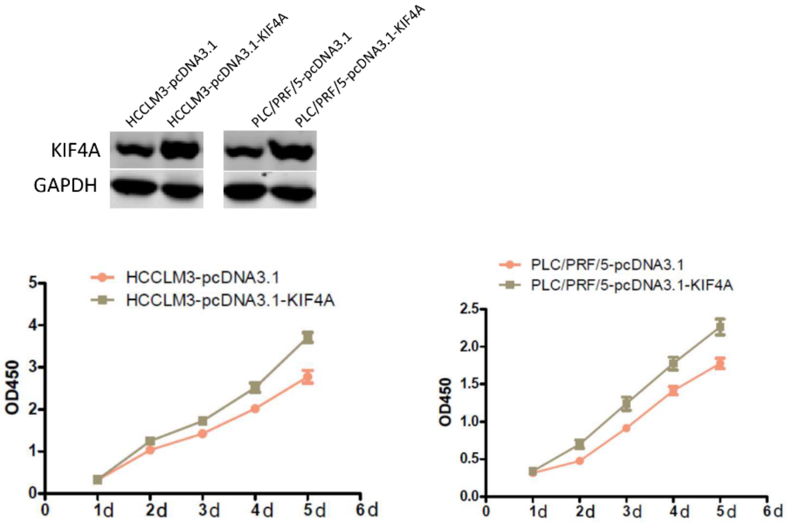

Supplementary Figure 2. The proliferation rate of HCC cell lines after overexpression of KIF4A using pc-DNA3.1-KIF4A (top) was enhanced compared to the control (Bottom left, HCCLM3 cell line; bottom right PLC/PRF/5 cell line). The y-axis indicates OD450 values, and x-axis indicates time points (in days).

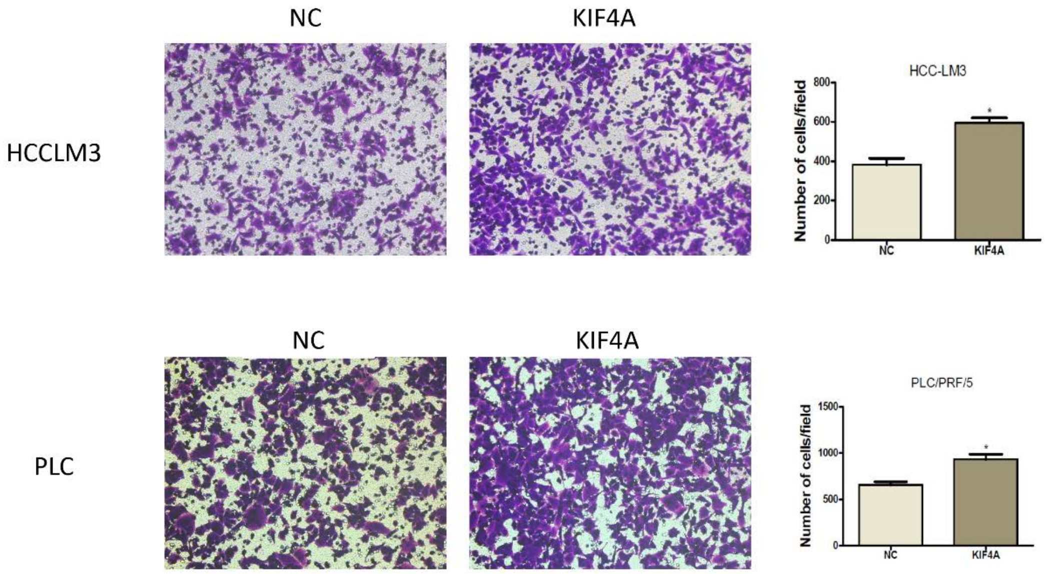

Supplementary Figure 3. The migration rate of HCC cell lines after overexpression of KIF4A using pc-DNA3.1-KIF4A was enhanced compared to the control (top, HCC-LM3 cell line; bottom, PLC/PRF/5 cell line).

Supplementary Table S1: Information of TCGA datasets used in this study.

Cancer Types	Number of sample	Overall survival	Number of Event
Adrenocortical Carcinoma	79	YES	27
Acute Myeloid Leukemia	173	YES	103
Brain Lower Grade Glioma	530	YES	92
Bladder Urothelial Carcinoma	407	YES	128
Breast Cancer	1102	YES	133
Cervical Squamous Cell Carcinoma	305	YES	70
Colorectal Adenocarcinoma	383	YES	70
Esophageal carcinoma	185	YES	69
Glioblastoma Multiforme	167	YES	114
Kidney Renal Clear Cell Carcinoma	534	YES	166
Kidney Renal Papillary Cell Carcinoma	291	YES	31
Liver Hepatocellular Carcinoma	371	YES	107
Lung Adenocarcinoma	513	YES	141
Lung Squamous Cell Carcinoma	502	YES	185
Stomach Adenocarcinoma	384	YES	122

Ovarian Serous Cystadenocarcinoma	266	YES	148
Pancreatic Adenocarcinoma	179	YES	83
Prostate Adenocarcinoma	498	NO	NA
Skin Cutaneous Melanoma	472	YES	200
Head and Neck Squamous Cell Carcinoma	521	YES	191
Sarcoma	262	YES	79
Thyroid Carcinoma	513	NO	NA
Testicular Germ Cell Cancer	156	NO	NA
Uterine Corpus Endometrioid Carcinoma	175	YES	23
Uterine Carcinoma	57	YES	33

Supplementary Table S2: KM survival and Cox regression analysis of KIF4A expression (median cutoff) on overall survival in patients across cancer types

Cancer Type	Log rank p	HR with 95% CI	p Value
ACC	4.41E-06	6.71(2.66 - 16.91)	5.39E-05
LAML	0.4309	0.85(0.58 - 1.26)	0.4239
BLCA	0.4242	1.15(0.81 - 1.63)	0.4256
BRAC	0.0031	1.68(1.19 - 2.39)	0.0034
CESC	0.3523	0.80(0.50 - 1.28)	0.3528
COADREAD	0.1942	0.73(0.45 - 1.17)	0.1954
ESCA	0.0303	1.70(1.05 - 2.77)	0.0322
GBM	0.9734	1.00(0.69 - 1.45)	0.9922
HNSC	0.5929	1.08(0.81 - 1.44)	0.5929
KIRC	4.90E-05	1.90(1.39 - 2.61)	6.49E-05
KIRP	0.0152	2.47(1.16 - 5.27)	0.0188
LGG	2.28E-05	2.73(1.68 - 4.43)	4.69E-05
LIHC	0.0004	1.87(1.32 - 2.67)	0.0005
LUAD	0.0056	1.60(1.14 - 2.25)	0.0061
LUSC	0.1898	0.82(0.62 - 1.10)	0.1908
OV	0.2027	1.23(0.89 - 1.71)	0.2041
PAAD	0.0024	1.97(1.26 - 3.08)	0.0028
SARC	0.4549	1.18(0.76 - 1.84)	0.456
SKCM	0.0128	1.43(1.08 - 1.89)	0.0133
STAD	0.9868	1.00(0.70 - 1.43)	0.9865
UCEC	0.0592	2.42(0.94 - 6.22)	0.0674
UCS	0.5998	0.83(0.41 - 1.67)	0.603
